# Editorial Changes

**Published:** 1849-02

**Authors:** 


*Editorial Changes. Medical Journals.*—Prof. Huston has resigned editorial connection with the *Medical Examiner,* and is succeeded by Francis G. Smith, M. D. and David H. Tucker, M. D. Prof. H. is one of the ablest medical critics our country affords, and, during his administration, the Examiner has been conducted with talent and independence. The new editors will have abundant cause for gratification with the success of their labors in maintaining the high character which the Journal has acquired.

By a copy of the *Cleveland Daily Herald,* which has been politely forwarded, we perceive that the first number of a new medical journal, entitled the *Western Medical Magazine and Review,* is to be issued on the first of the present month. It is to be edited by J. J. Delamater, M. D., Prof. of Anat. and Phys. in the Cleveland Medical College, assisted by his colleagues as collaborators. It is to be a bi-monthly of 128 pages. We trust that the editor may find in his editorial duties, an agreeable relaxation from the labor and cares incident to his professorial and professional avocations. With such an expectation probably many undertakings of the kind have been commenced; how fully it may be realized, experience will reveal to Dr. D., as it has to those who have anticipated him in a similar experiment. We heartily wish the effort success, extending, as in courtesy bound, to a newly acquired brother of the *corps editoriale,* the hand of fraternal fellowship.

The able editor of the *Southern Medical and Surgical Journal,* Professor Paul F. Eve, has given notice that the Journal will be discontinued after the present year, owing to want of adequate patronage. He states that the privilege of laboring for his readers during the past year, has required, from his own pocket, an outlay of $900, and he very reasonably concludes that the luxury of serving the medical public on these terms is too expensive to be longer indulged. That a periodical of such merit as the Southern Medical and Surgical Journal should require more than the gratuitous services of the editor to secure ample support, is a stigma upon our southern brethren which we sincerely hope they will not consent to bear. We anticipate that the effect of the notice referred to, will be to secure a list of *paying* subscribers, that will not only justify the continuance of the work, but furnish a complimentary testimonial to the value of the editor's past labors.

The *Western Journal of Medicine and Surgery* recently annnounced that the work would cease, unless subscribers at once signified their wish for its continuance by paying their dues. We are happy to perceive by the last number of our esteemed contemporary, that the required conditions have been complied with.

Rotation in office, it would seem, is a rule of medical editorship, as of politics, in this country. Since our Journal was commenced, a little less than four years ago, fourteen brethren of the *corps* have retired. This fact would imply either that the honors and emoluments of editorial office are so abundant that incumbents are soon satisfied, or else, quite the reverse. We will give an opinion as to which supposition is the most probable, when we conclude, as a Journalist, to make our "dying speech and confession."

